# *KRAS* A146 Mutations Are Associated With Distinct Clinical Behavior in Patients With Colorectal Liver Metastases

**DOI:** 10.1200/PO.21.00223

**Published:** 2021-11-17

**Authors:** Iris van 't Erve, Nina J. Wesdorp, Jamie E. Medina, Leonardo Ferreira, Alessandro Leal, Joost Huiskens, Karen Bolhuis, Jan-Hein T. M. van Waesberghe, Rutger-Jan Swijnenburg, Daan van den Broek, Victor E. Velculescu, Geert Kazemier, Cornelis J. A. Punt, Gerrit A. Meijer, Remond J. A. Fijneman

**Affiliations:** ^1^ Department of Pathology, The Netherlands Cancer Institute, Amsterdam, the Netherlands; ^2^ Deparment of Surgery, Cancer Center Amsterdam, Amsterdam University Medical Centers, VU University, Amsterdam, the Netherlands; ^3^ Sidney Kimmel Comprehensive Cancer Center, Johns Hopkins University School of Medicine, Baltimore, MD; ^4^ Center for Personalized Medicine, Hospital Israelita Albert Einstein, São Paulo, Brazil; ^5^ SAS Institute B.V., Huizen, the Netherlands; ^6^ Department of Medical Oncology, Amsterdam University Medical Centers, University of Amsterdam, Amsterdam, the Netherlands; ^7^ Deparment of Radiology and Molecular Imaging, Cancer Center Amsterdam, Amsterdam University Medical Centers, VU University, Amsterdam, the Netherlands; ^8^ Department for Laboratory Medicine, The Netherlands Cancer Institute, Amsterdam, the Netherlands; ^9^ Julius Center for Health Sciences and Primary Care, University Medical Center Utrecht, Utrecht, the Netherlands

## Abstract

Somatic *KRAS* mutations occur in approximately half of the patients with metastatic colorectal cancer (mCRC). Biologic tumor characteristics differ on the basis of the *KRAS* mutation variant. *KRAS* mutations are known to influence patient prognosis and are used as predictive biomarker for treatment decisions. This study examined clinical features of patients with mCRC with a somatic mutation in *KRAS* G12, G13, Q61, K117, or A146.

**METHODS:**

A total of 419 patients with colorectal cancer with initially unresectable liver-limited metastases, who participated in a multicenter prospective trial, were evaluated for tumor tissue *KRAS* mutation status. For the subgroup of patients who carried a *KRAS* mutation and were treated with bevacizumab and doublet or triplet chemotherapy (N = 156), pretreatment circulating tumor DNA levels were analyzed, and total tumor volume (TTV) was quantified on the pretreatment computed tomography images.

**RESULTS:**

Most patients carried a *KRAS* G12 mutation (N = 112), followed by mutations in G13 (N = 15), A146 (N = 12), Q61 (N = 9), and K117 (N = 5). High plasma circulating tumor DNA levels were observed for patients carrying a *KRAS* A146 mutation versus those with a *KRAS* G12 mutation, with median mutant allele frequencies of 48% versus 19%, respectively. Radiologic TTV revealed this difference to be associated with a higher tumor load in patients harboring a *KRAS* A146 mutation (median TTV 672 cm^3^ [A146] *v* 74 cm^3^ [G12], *P* = .036). Moreover, *KRAS* A146 mutation carriers showed inferior overall survival compared with patients with mutations in *KRAS* G12 (median 10.7 *v* 26.4 months; hazard ratio = 2.5; *P* = .003).

**CONCLUSION:**

Patients with mCRC with a *KRAS* A146 mutation represent a distinct molecular subgroup of patients with higher tumor burden and worse clinical outcomes, who might benefit from more intensive treatments. These results highlight the importance of testing colorectal cancer for all *KRAS* mutations in routine clinical care.

## INTRODUCTION

Oncogenic *KRAS* mutations are highly prevalent in multiple cancers and drive cell differentiation and proliferation.^1^ *KRAS* mutations stimulate KRAS to stay in its active state, thereby triggering the oncogenic signaling pathway.^2^ Around 40%-50% of the patients with metastatic colorectal cancer (mCRC) harbor a somatic *KRAS* mutation.^3-5^ In general, patients with a *KRAS* wild-type tumor have a better prognosis than patients carrying a *KRAS*-mutated tumor.^6,7^ Moreover, *KRAS* mutation status is a predictive marker for poor response to anti–epidermal growth factor receptor (EGFR) monoclonal antibody therapy,^8^ one of the options for systemic treatment for patients with mCRC.^9^ Therefore, analysis of *KRAS* mutation status has been widely adopted in routine clinical practice.^10^

**CONTEXT**

**Key Objective**
The distribution of *KRAS* mutation variants across tumor types is not uniform. The *KRAS* A146 mutation is predominantly seen in patients with colorectal cancer. Here, we evaluated how clinical features like tumor load and overall survival differ between patients with metastatic colorectal cancer (mCRC) carrying distinct somatic *KRAS* G12, G13, Q61, K117, or A146 mutations.
**Knowledge Generated**
This study revealed that within patients with mCRC, A146 is the third most common *KRAS* mutation variant. Patients with mCRC with a *KRAS* A146–mutated tumor represent a distinct molecular subgroup of patients with higher tumor burden that is associated with worse clinical outcomes.
**Relevance**
These results highlight the clinical importance of testing colorectal cancer for all *KRAS* mutations in routine diagnostics. The distinct clinical implications of *KRAS* A146 mutations in patients with mCRC warrant further investigation regarding therapeutic strategies to target and treat *KRAS* A146 mutant tumors.

It is known that the biologic characteristics of tumors, like cellular phenotypes and metabolomic characteristics, differ on the basis of the *KRAS* mutation variant and amino acid substitution.^11-13^ In a substantial part of routine diagnostic *KRAS* tissue panels, only the most common driver mutations in *KRAS* codons G12 and G13 are tested, which are affected in 28% and 8% of all patients with mCRC, respectively. However, mutations are also commonly present in *KRAS* Q61 (2%), K117 (1%), and A146 (4%).^4,5,14^ Here, we investigated clinical features like tumor load and overall survival of patients with mCRC with a somatic mutation in *KRAS* G12, G13, Q61, K117, or A146.

## METHODS

### Patient Characteristics

Liquid biopsies of patients with histologically proven colorectal cancer (CRC) with isolated, previously untreated, initially unresectable colorectal liver metastases (CRLM) were collected in the ongoing multicenter phase III CAIRO5 trial (NCT02162563).^15^ A total of 419 patients with CRLM, enrolled between November 2014 and July 2019, were evaluated in this study. For all patients, tissue *KRAS* mutation analyses were performed in the participating hospitals before randomization following routine clinical practice. Only those patients who were randomized for treatment with bevacizumab and chemotherapy consisting of 5-fluorouracil, leucovorin, oxaliplatin, and/or irinotecan were selected for the current study. Clinical follow-up was performed according to standard of care, including clinical review every three months as well as computed tomography (CT) imaging every six months, for patients with resectable disease and every two months for patients with unresectable disease, using the RECIST 1.1 for reporting. Follow-up was recorded until March 23, 2020. The study was performed in accordance with the Declaration of Helsinki and a medical ethical committee approved the trial, and all patients signed written informed consent for study participation and liquid biopsy collection.

### Liquid Biopsy Collection

Liquid biopsies were collected before study treatment using a cell-stabilizing BCT tube (Streck, La Vista, NE) in the participating hospitals and shipped to the Netherlands Cancer Institute. Here, cell-free plasma was collected in a two-step centrifugation process: 10 minutes at 1.700 g followed by 10 minutes at 20.000 g, and stored at –80°C until further processing. Cell-free DNA (cfDNA) was isolated using the QIAsymphony (Qiagen, Hilden, Germany) with an elution volume set to 60 µL. The concentration of cfDNA was measured using the Qubit dsDNA High-Sensitivity Assay (TFS, Waltham, MA).

### Liquid Biopsy Mutation Analyses

For patients with an established *KRAS* mutation on the basis of tumor analysis, liquid biopsy mutation analyses were performed using four droplet digital polymerase chain reaction (ddPCR; Bio-Rad, Hercules, CA) screening kits, namely ddPCR KRAS G12/G13 (#1863506), ddPCR KRAS Q61 (#12001626), ddPCR KRAS K117N (#10049047), and ddPCR KRAS A146T (#10049550). Table 1 in the Data Supplement shows the different amino acid variants detected by these assays. The ddPCR assays were performed according to the manufacturer's instruction, making use of 1 µL of the multiplex assay, 11 µL of the ddPCR supermix for probes (no dUTP), 9 µL of sample, and 1 µL H_2_O. All measurements were performed in duplicate and included a blank (nuclease-free water) and a positive control. Data were analyzed using the QuantaSoft software version 1.6.6 (Bio-Rad, Hercules, CA). The number of mutant copies per mL plasma (MTc/mL) and mutant allele frequency (MAF) were used as outcome measures. For the cfDNA samples with a *KRAS* A146 mutation, orthogonal validation was performed using targeted deep sequencing, as described previously.^16^ In brief, genomic libraries were prepared from 125 ng of cfDNA, following normalization, end-repair, A-tailing, adapter ligation, and PCR amplification. Target capture was performed using a panel consisting of 58 genes, covering 81 kb. Candidate somatic alterations across the region of interest were identified using VariantDx (Personal Genome Diagnostics, Baltimore, MD).

### Radiologic Total Tumor Volume Quantification

For patients with an identified *KRAS* mutation on tumor tissue, pretreatment contrast-enhanced abdominal CT images were used for semiautomatic segmentation in the Tumor Tracking Modality of IntelliSpace Portal 9.0 (Philips, Eindhoven, the Netherlands). The liver itself and all metastases were segmented by two trained members of the research team and subsequently adjusted and verified by a radiologist specialized in abdominal pathology. All segmentations and related CT images were processed and analyzed with the SAS Viya analytical platform (SAS Institute Inc, Cary, NC) for volume quantification using the quantifyBioMed Images action.^17^ This action calculates the total tumor volume (TTV) directly out of the segmentation from all tumors presented in the liver by determining the volume of one voxel and multiplying this volume with the number of voxels included in the tumor segmentation. A CT scan is built up by voxels, the three-dimensional equivalent of a pixel, and a voxel's volume depends on the pixel spacing and slice thickness attributes of the CT scan. The volume of the liver was calculated similarly, on the basis of the three-dimensional liver segmentation. In addition, the percentage TTV of the total liver volume, including TTV, was calculated. Furthermore, the radiologist registered the number of liver lesions.

### Statistical Analyses

A Brown–Forsythe analysis of variance test using Dunnett's multiple comparisons was used for the liquid biopsy analyses. A one-way analysis of variance corrected for multiple comparisons using Tukey's multiple comparisons test was used for the volumetric analyses. A two-sided *P*-value of .05 was used as a cutoff for significance. A Mantel–Cox log-rank test using a Bonferroni-corrected threshold of *P* < .005 for significance was performed for the survival analyses. To determine the equivalence between ddPCR and sequencing circulating tumor DNA (ctDNA) levels, a Pearson correlation was used. Clinical patient characteristics were compared between carriers of different *KRAS* mutant variants using Fisher's exact tests. Univariate and multivariate Cox proportional hazards regression analyses were performed to analyze prognostic factors for overall survival, adjusted for potential confounders. Analyses were performed with Prism version 8 (GraphPad Software, Inc, San Diego, CA) and SPSS software version 27 (IBM, New York, NY).

## RESULTS

### Patient Characteristics

**FIG 1.**
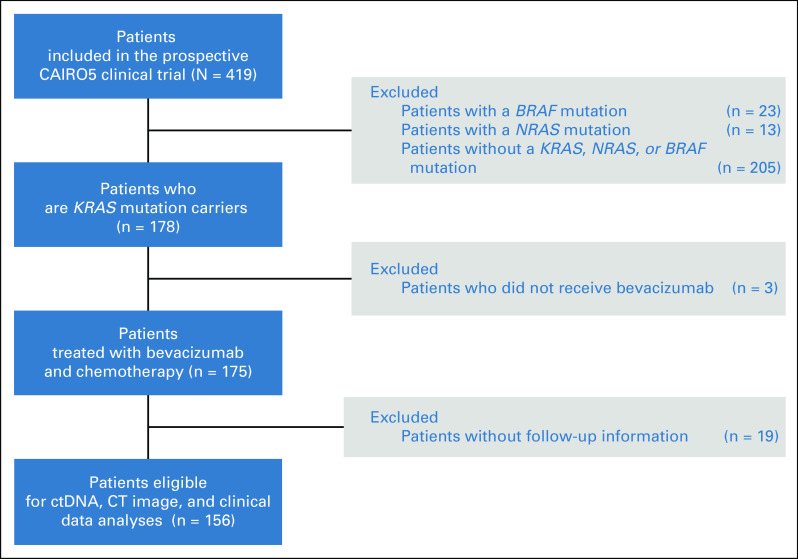
Flowchart of patient selection. *KRAS*, *NRAS*, and *BRAF* mutation status was determined on tumor tissue for a total of 419 patients with CRC with isolated and initially unresectable liver metastases enrolled in the CAIRO5 clinical trial. Patients without a *KRAS*-mutated tumor were excluded from the current study. Next, patients not treated with bevacizumab and chemotherapy were excluded to ensure a homogenous patient group. Last, patients without clinical follow-up were excluded, resulting in 156 patients with mCRC for analyses. CRC, colorectal cancer; ctDNA, circulating tumor DNA; CT, computed tomography; mCRC; metastatic colorectal cancer.

**FIG 2.**
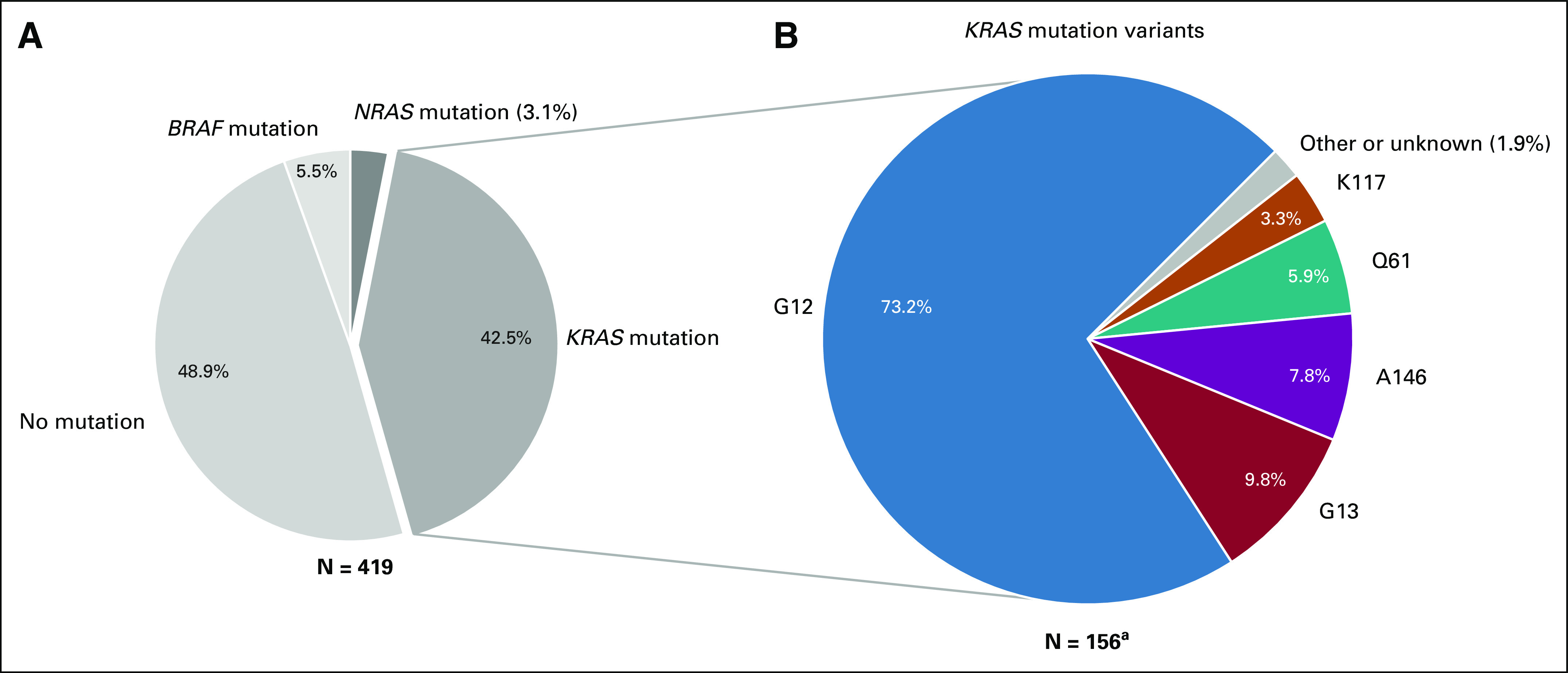
(A) The distribution of *KRAS/NRAS/BRAF* mutations among the 419 patients with CRLM evaluated in this study and (B) the relative distribution of *KRAS* codon variants among 156 *KRAS* mutation carriers. ^a^Of note, 42.5% of the 419 patients (N = 178) carried a tumor tissue *KRAS* mutation, of whom 156 patients were used for further analyses (see Fig 1). CRLM, colorectal liver metastases.

Of the 419 patients evaluated, 178 patients (42%) met the selection criteria and carried a tumor tissue *KRAS* mutation. Three patients who did not receive bevacizumab and 19 patients unavailable for follow-up were excluded, leaving 156 patients for analyses (Fig 1). The majority of these patients carried a *KRAS* G12 mutation (N = 112, 71.8%), followed by mutations in G13 (N = 15, 9.6%), A146 (N = 12, 7.7%), Q61 (N = 9, 5.8%), K117 (N = 5, 3.2%), and A59 (N = 1, 0.6%). The codon affected was unknown for two patients (1.3%; Fig 2). Clinical patient characteristics per *KRAS* mutation and per *KRAS* most frequent G12 residues (G12A, G12C, G12D, and G12V) are shown in Table 1 and Table 2 in the Data Supplement, respectively.

**TABLE 1.**
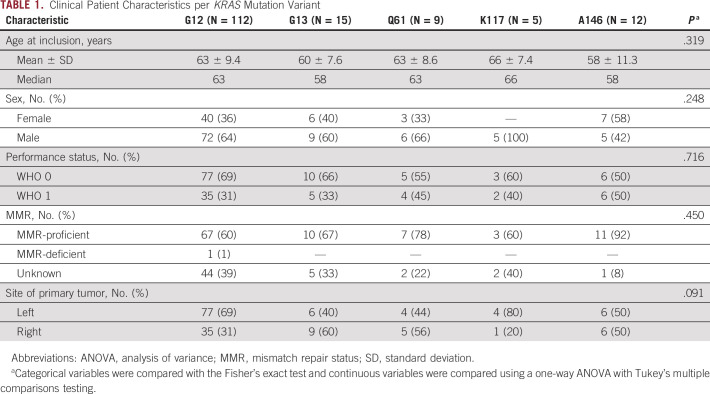
Clinical Patient Characteristics per *KRAS* Mutation Variant

### High Plasma ctDNA Levels in Patients With *KRAS* A146 Mutant Tumors

**FIG 3.**
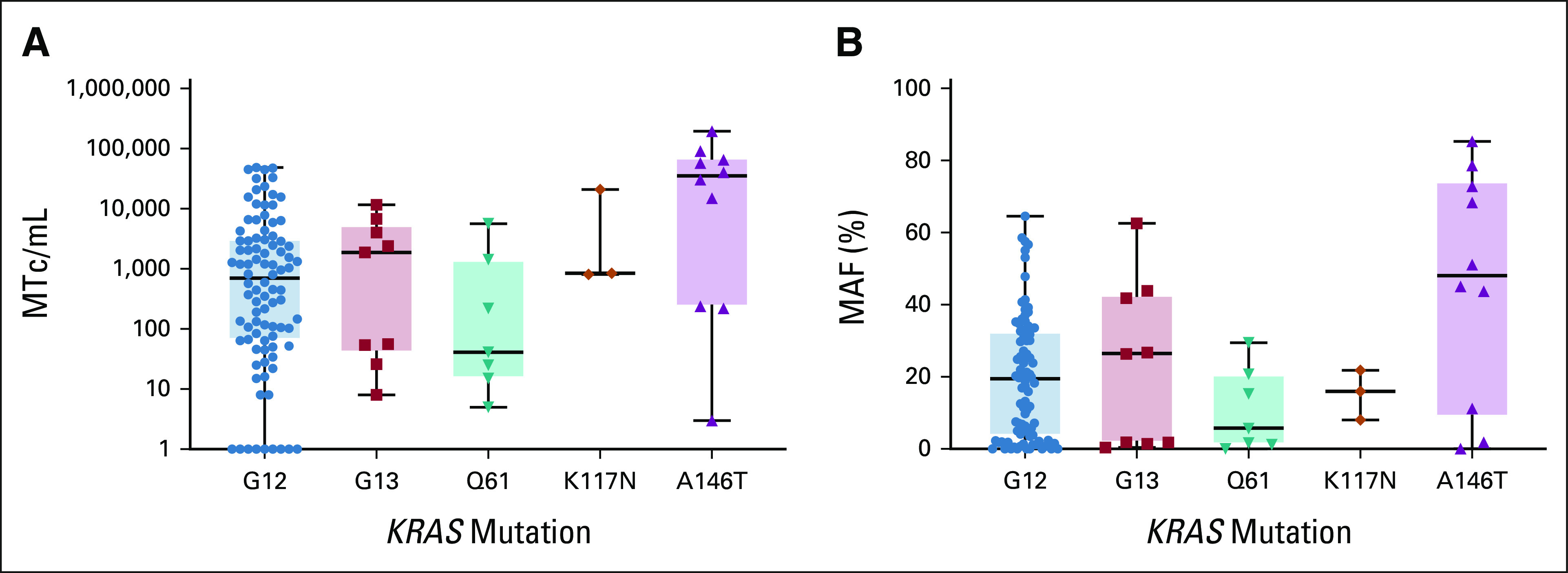
(A) MTc/mL plasma and (B) MAF detected by ddPCR analyses of pretreatment liquid biopsies stratified per *KRAS* mutation variant. No significant differences were observed upon Brown-Forsythe ANOVA and Dunnett's multiple comparison testing. ANOVA, analysis of variance; ddPCR, droplet digital polymerase chain reaction; MAF, mutant allele frequency; MTc/mL, mutant copies per mL.

We previously measured plasma ctDNA levels in 100 patients with CRLM and noticed remarkably high plasma ctDNA levels in patients harboring a *KRAS* A146–mutated tumor, an observation that warranted further investigation.^18^ The current study investigated the liquid biopsy ctDNA levels for all 156 patients included. Patients without a pretreatment liquid biopsy (N = 32) and patients carrying a tumor with a *KRAS* mutation that could not be detected by the ddPCR kits (N = 2) were excluded, leaving 122 ctDNA samples for liquid biopsy analyses (Data Supplement Figure 1). Liquid biopsy ddPCR analyses showed more MTc/mL plasma and a higher MAF for patients with *KRAS* A146–mutated tumors (N = 10, median MTc/mL = 35,338, median MAF = 48%) compared with patients carrying a different *KRAS* variant, for example, a *KRAS* G12 mutation (N = 92, median MTc/mL = 700, median MAF = 19%), see Figure 3A (MTc/mL) and Figure 3B (MAF). To ensure that these high plasma ctDNA levels were not because of the *KRAS* codon 146 ddPCR assay's test characteristics, we performed orthogonal testing using a targeted deep-sequencing approach. A strong confirmation of the high *KRAS* A146 ctDNA levels was observed, with a Pearson correlation (*R*^2^) of 0.98 (95% CI, 0.96 to 1.00; *P* < .0001) between the ddPCR and sequencing MAF results (Figure 2 in Data Supplement). The high plasma ctDNA levels in *KRAS* A146 mutation carriers were not caused by DNA copy-number gains or focal amplification of the *KRAS* locus (see methods in Data Supplement). Moreover, the high plasma ctDNA levels in patients harboring a *KRAS* A146–mutated tumor were accompanied by high plasma ctDNA levels for other genes like *TP53*, *TERT*, and *PIK3CA* (Figure 3 in Data Supplement), implying that high plasma ctDNA levels for *KRAS* A146–mutated tumors are associated with tumor burden.

### Patients With *KRAS* A146–Mutated Tumors Have High TTV

**FIG 4.**
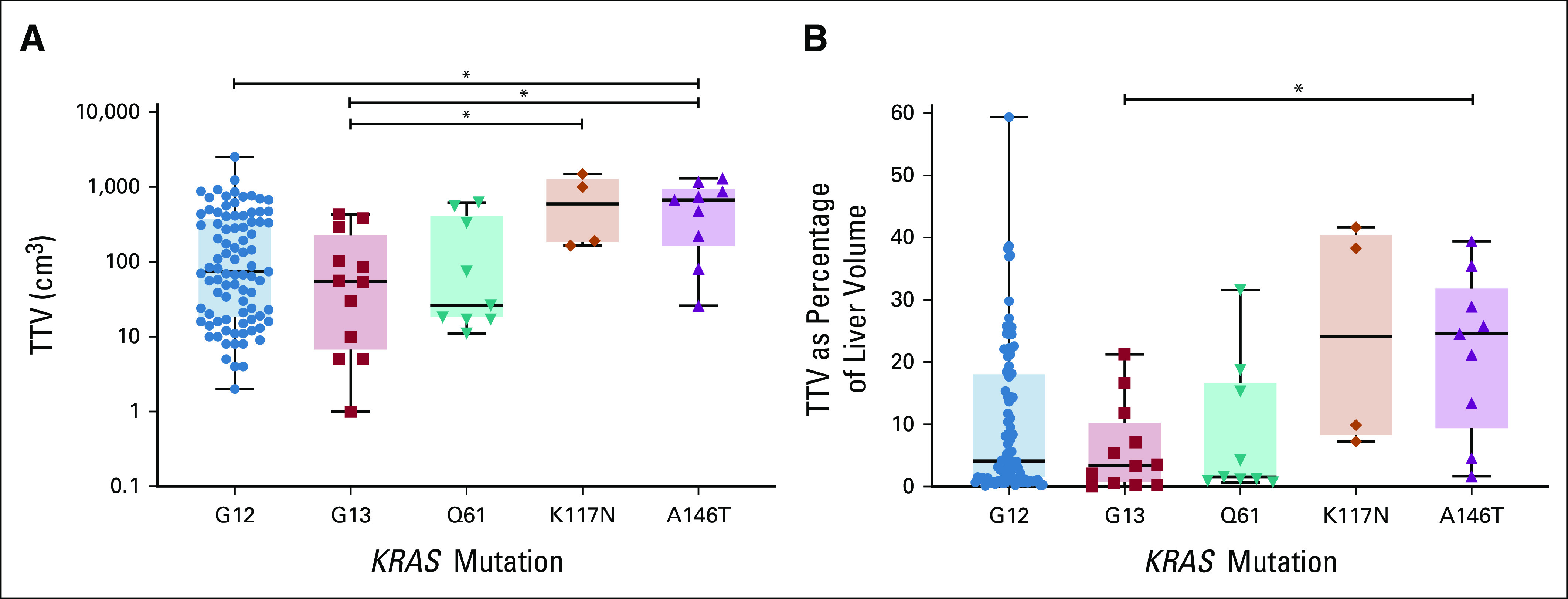
(A) The absolute TTV in cm^3^ and (B) the relative TTV as a percentage of the liver volume per *KRAS* mutation variant assessed using volumetric analyses of pretreatment CT imaging. *P < .05; one-way ANOVA; Tukey's multiple comparisons test. ANOVA, analysis of variance; CT, computed tomography; TTV, total tumor volume.

**FIG 5.**
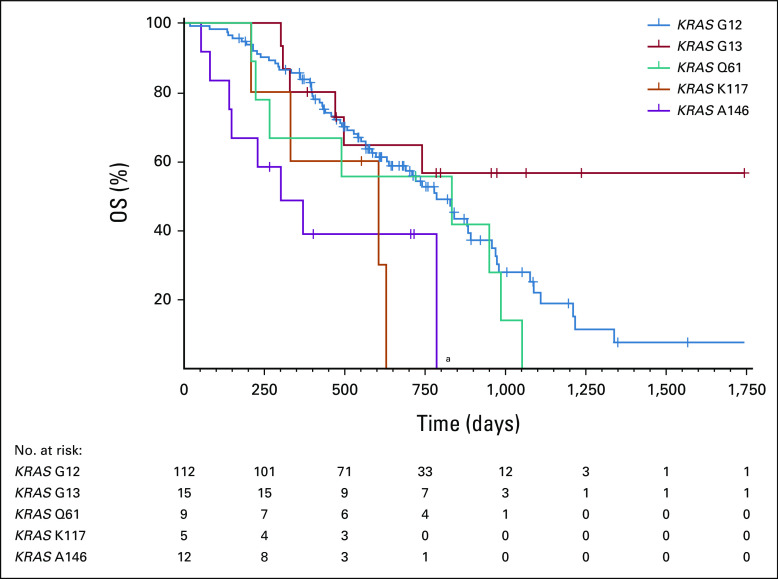
Overall survival of patients with mCRC carrying a tumor with a *KRAS* G12, G13, Q61, K117, or A146 mutation. Median survival in months was 26.4 (*KRAS* G12), undefined (*KRAS* G13), 27.9 (*KRAS* Q61), 20.6 (*KRAS* K117), and 10.7 (*KRAS* A146). ^a^A significant difference was observed between *KRAS* G12 and A146 (log-rank *P* = .0045), by the Mantel-Cox log-rank test using a Bonferroni-corrected threshold for every combination of *P* < .005 for significance. mCRC, metastatic colorectal cancer.

As all patients in this study had liver-only metastases, total tumor burden could be assessed by measuring the pretreatment TTV. Since abdominal contrast-enhanced CT images could be used for segmentation, patients with a magnetic resonance imaging (N = 17) and positron emission tomography-CT (PET-CT) or non–contrast-enhanced scans (N = 4) were excluded from the volumetric analysis. Other reasons for exclusion were technical errors in the segmentation software (N = 3), missing scans (N = 2), and incomplete scans (N = 4), leaving 126 patients for volumetric analysis. Figure 4A shows the absolute TTV and Figure 4B shows the relative TTV as percentage of the liver volume. Patients with a *KRAS* A146–mutated tumor have a significantly higher absolute and relative TTV (median TTV of 672 cm^3^ and 24.5% of total liver volume) compared with patients with a *KRAS* G12 mutation (median TTV of 74 cm^3^ and 4.1% of total liver volume; *P* = .036 and *P* = .053, respectively) and G13 mutation (median TTV of 55 cm^3^ and 3.5% of total liver volume; *P* = .021 and *P* = .026, respectively). In addition, the median number of lesions tended to be higher for patients with *KRAS* A146 mutant tumors (median = 25) compared with patients with one of the other *KRAS* variants (median number of lesions *KRAS* G12 = 11, G13 = 10.5, Q61 = 10, K117 = 14; see Figure 4 in the Data Supplement). High TTV was also observed in patients with the less prevalent *KRAS* K117 mutation (median absolute TTV = 592 cm^3^, relative TTV = 24.1%). The volumetric results of the four most frequent G12-mutated residues (G12A, G12C, G12D, and G12V) did not differ significantly (Figure 5 in Data Supplement).

### *KRAS* A146–Mutated Tumors Are Associated With Poor Overall Survival

Patients with mCRC with a *KRAS* A146–mutated tumor showed a worse prognosis than patients with another *KRAS* mutation variant (Fig 5). The overall survival of patients with a *KRAS* A146–mutated tumor was significantly shorter compared with patients with a *KRAS* G12–mutated tumor (median 10.7 *v* 26.4 months). Also, patients with the less common *KRAS* K117 mutation progressed faster, whereas patients with a tumor with a mutation in *KRAS* G13 had the most favorable prognosis. Univariable Cox regression analyses showed that age, sex, sidedness of the primary tumor, and WHO performance status were not associated with overall survival (Table 3 in Data Supplement). After adjusting for these clinical characteristics (age, sex, sidedness, and performance status), the multivariable Cox regression analysis showed that only the *KRAS* alteration was an independent prognostic factor for worse overall survival. The reported contrast between the *KRAS* mutation variants showed that *KRAS* A146 was the only significant feature behind this observation (hazard ratio = 2.5; 95% CI, 1.4 to 4.6; log-rank *P* = .003). No indications of an association between the baseline patient characteristics and the *KRAS* mutation variants were found (Table 1). No significant differences were seen in overall survival between the four most frequently mutated G12 residues (G12A, G12C, G12D, and G12V; see Figure 6 in the Data Supplement). Furthermore, the location of disease progression showed similar patterns for the different *KRAS* mutation variants (Figure 7 in Data Supplement).

## DISCUSSION

Oncogenic *KRAS* mutations occur in approximately 50% of patients with mCRC and are known to be predictive for treatment response and to affect patient prognosis.^10,11,19^ The level of *KRAS* oncogene activation can vary depending on the amino acid change, resulting in different biologic and clinical behavior.^11,20^ Here, we studied the clinical impact of *KRAS* mutation variants in a homogenous group of patients with CRLM and demonstrated that patients with CRLM with a *KRAS* A146–mutated tumor have a high tumor load, which was associated with inferior survival compared with patients with other *KRAS* mutations.

The observations made in this study cannot directly be translated to other cancer types, since the behavior of *RAS* mutation variants is dependent on the location and cell type of the tumor.^11^ For example, the large number of samples analyzed and reported in cBioPortal^21^ show that *KRAS* A146 mutations are rarely reported in other cancer types except for CRC.^21-23^ Although the prevalence of A146 mutations among *KRAS* mutation carriers in CRC is approximately 8%, similar to our observation in this study, it is only 0%-0.5% in both lung^24-26^ and pancreas cancer.^27,28^

Biologically, *KRAS* mutation variants display distinct metabolic profiles. Oncogenic KRAS can dysregulate cell metabolism via glycolysis and the following tricarboxylic acid cycle. Enhanced glycolysis of cancer cells generating lactate even when exposed to abundant oxygen (Warburg effect)^29^ is shown to be upregulated via oncogenic KRAS.^30-32^ This Warburg effect is marked by low levels of ATP. However, a human CRC cell line study revealed distinct metabolic profiles of different *KRAS* mutation variants. Where for most *KRAS* variants the nucleotide imbalance is shifted toward a decrease in ATP and other nucleotides like guanosine triphosphate (GTP), cell lines harboring the *KRAS* A146 mutation displayed increased levels of these nucleotides.^12^ The distinct metabolic profiles among *KRAS* mutation variants do not directly explain the observed differences in clinical outcome. Future research is needed to examine whether *KRAS* A146–mutated tumors are a distinct metabolic subgroup for which other therapeutic targets might be beneficial.

The differences in biology and consequently clinical outcome between *KRAS* mutation variants observed in this study might originate from the molecular mechanism of oncogenic activation. KRAS changes between two nucleotide-binding states, the inactive form (guanosine diphosphate–bound) and the active (GTP-bound) form, with the help of guanine nucleotide exchange factors (GEF) and GTPase-activating proteins (GAP). Somatic mutations stimulate KRAS to be in the active GTP-bound form,^33^ but the impairment of GTP hydrolysis occurs via different mechanisms.^11,34^ *KRAS* G12 and Q61 mutations mainly affect GAP-driven GTP hydrolysis, whereas mutations in G13 and K117 influence both GEF and GAP.^35,36^ By contrast, mutations in *KRAS* A146 cause an increase in GEF-mediated nucleotide exchange without affecting GAP activity,^37^ suggesting that tumors with a *KRAS* A146 mutation may be prone to respond to GEF inhibitors. Inhibition of the GEF Son Of Sevenless protein 1 (SOS1) reduces KRAS activation, especially when combined with an MEK inhibitor.^38^ Likewise, RAS activation via GEFs was reduced by inhibition of the protein tyrosine phosphatase SHP2,^39^ which was more effective in cells harboring *KRAS* G12C compared with cells harboring *KRAS* G12D.^40^ Recently, AMG 510 (sotorasib), an antitumor agent targeting *KRAS* G12C mutant advanced solid tumors, has shown to improve the efficacy of (targeted) treatments in vivo.^41^ AMG 510 is currently under investigation in a clinical trial (NCT03600883), including patients with CRC and non–small-cell lung cancer.^42^ Another potential treatment strategy could be dual phosphatidylinositol 3-kinase (PI3K)/mammalian target of rapamycin (mTOR) inhibition. Overexpression of the PI3K/Akt/mTOR signaling pathway is common in (m)CRC, resulting in enhanced tumor growth. Dual PI3K/mTOR inhibitors have shown to reduce cell proliferation of *PIK3CA* mutant tumors in mice^43^ and phase I clinical studies.^44^ However, this effect was not seen in cell lines where *KRAS* and *PIK3CA* mutations co-occurred.^45^ When combining the dual PI3K/mTOR inhibitor with an MEK inhibitor, significant tumor reduction was seen in *KRAS* mutant tumors.^46,47^ No data are available on combined PI3K/mTOR and MEK inhibition for patients with *KRAS* A146 mCRC specifically. The high tumor burden observed in our study makes the PI3K/mTOR signaling pathway an interesting potential druggable target for patients with *KRAS* A146–mutated tumors. Future research is needed to find out whether PI3K/mTOR inhibition combined with an MEK inhibitor has potential for *KRAS* A146–mutated tumor and if the poor long-term tolerability found in other advanced solid tumors^48^ is also pertinent in patients with *KRAS* A146 mutant tumors. Taken together, these results show promising potential for therapeutic targeting of *KRAS* mutation variants and warrant further investigation regarding therapeutic strategies to specifically target tumors with a *KRAS* A146 mutation.

A better insight into the *KRAS* mutation status can help guide and personalize the treatment approach of patients with mCRC. Previous studies in patients with early-stage CRC and CRLM showed worse outcomes for patients with a *KRAS* G12V,^49-53^ G12C,^50-53^ or G12S^49^ tumor mutation compared with other frequently occurring G12 variants, like G12A and G12D. In our study, patients with *KRAS* G12C and G12V mutations tended to have inferior survival compared with *KRAS* G12D and G12A mutations. Interestingly, in contrast to the current study investigating patients with CRC in the metastatic setting, patients with nonmetastatic CRC with a *KRAS* A146–mutated tumor showed better survival compared with patients with mutations in other *KRAS* codons.^4,54^ Whereas the patients with early-stage CRC carrying a *KRAS* A146 mutation in the study of Janakiraman et al had more frequent KRAS copy-number gains, we did not observe such copy-number aberrations in the patients with *KRAS* A146 mutant mCRC. The location and extent of metastases might also influence survival differences. The focus on unresectable liver-only metastases is specific for this study. Furthermore, the biologic characteristics of tumors might differ on the basis of the specific *KRAS* A146 amino acid substitution, similar to the differences observed between *KRAS* G12 variants.

In current clinical practice, no distinction is made based between *KRAS* mutation variants with regards to anti-EGFR treatment. However, data on the effect of anti-EGFR treatment in patients with CRC with a *KRAS* A146 tumor mutation are conflicting. Some studies describe a more favorable clinical outcome in patients with CRC with a *KRAS* A146-mutated tumor upon anti-EGFR treatment compared with patients with tumors carrying another *KRAS* mutation.^54-57^ Other studies show that tumors with a *KRAS* A146 mutation, like other *KRAS* mutations, are responsible for anti-EGFR resistance.^58-61^In this study, the homogenous population of initially unresectable liver-only metastatic CRC patients all receiving the same treatment regimen allowed for an unbiased comparison of the clinical features of patients harboring different *KRAS* tumor mutations. Another strength of this study is the objective assessment of tumor burden by TTV quantification on the basis of semiautomatic segmentations of the tumor. However, the assessment of TTV is not implemented in clinical practice, as it remains time-consuming and advanced volumetric software is not yet widely available in every radiology department. Since *KRAS* A146 mutations occur in roughly 4% of patients with mCRC, only a limited number of patients were available with a *KRAS* A146–mutated tumor despite the large number of patients included in the clinical trial. The percentage of patients with a *KRAS* A146–mutated tumor might be even higher than depicted in this study because routine molecular diagnostics is sometimes limited to the most common *KRAS* driver mutations, that is, the G12 and G13 variants. Although *KRAS* Q61, K117, and A146 mutations occur less frequently, this study indicates it is important to implement *KRAS* mutation testing for all variants (G12, G13, Q61, K117, and A146) in routine diagnostics.^8,59,62^

In conclusion, patients with mCRC with a *KRAS* A146 mutation represent a distinct molecular subtype of patients with poor survival who might benefit from more intensive treatments. Therefore, *KRAS* A146 mutation testing should be adopted in routine diagnostic testing.
